# Recommendations for physiotherapy and physical activity for children with Legg–Calvé–Perthes disease: a survey of pediatric orthopedic surgeons and physiotherapists in Sweden

**DOI:** 10.2340/17453674.2023.18341

**Published:** 2023-08-16

**Authors:** Louise MELIN, Zlatica RENDEK, Yasmin D HAILER

**Affiliations:** Section of Orthopedics, Department of Surgical Sciences, Uppsala University Hospital, Uppsala, Sweden

## Abstract

**Background and purpose:**

Physiotherapy, restrictions of physical activity, and weightbearing are part of the treatment of children with Legg–Calvé–Perthes disease (LCPD). Prescription practices are widely discussed and vary between pediatric orthopedic surgeons (POSs) and physiotherapists (PTs). The purpose of this study was to identify recommendations for treatment methods in clinical practice to find some consensus and elaborate guidelines.

**Patients and methods:**

A web-based questionnaire including 3 cases of LCPD (initial, fragmentation, and reossification stages) was answered by 25 POSs and 19 PTs. They were asked to describe their preferred recommendations for physiotherapy, including stretching, strengthening, weightbearing, and physical activities in relation to, e.g., range of motion (ROM) pain, sex, and disease stage.

**Results:**

ROM was considered to be important when recommending physiotherapy; PTs also recognized pain and disease stage. Sex was reported as a factor with low importance. Stretching exercises were recommended for all disease stages. Recommendations for strengthening exercises varied for the initial and fragmentation stages. None of the participants recommended total non-weightbearing. Most restricted trampolining, running, ball sports, and gymnastics in the first 2 stages of the disease and allowed swimming, short walks, cycling, and horse riding without restrictions for all stages.

**Conclusion:**

We found high agreement on recommending stretching exercises for all disease stages, but controversies regarding recommendations for strengthening exercises in the initial and fragmentation stages. No non-weightbearing treatment for the affected hip was recommended by any participants at any stage of the disease. There was no clear consensus regarding the appropriate timeline for resuming full activities.

Legg–Calvé–Perthes disease (LCPD) is a pediatric hip disorder caused by ischemic necrosis of the femoral head [1,2]. There is no consensus regarding the optimal non-surgical treatment consisting of physiotherapy (stretching and strengthening) and physical activity [3].

Early in the pathologic process, the femoral head is vulnerable to mechanical forces, which might be responsible for its deformation [4,5] and weight loading may cause flattening and widening of the femoral head in the early stages of LCPD [6].

Physiotherapy has been shown to improve articular range of motion (ROM) [7] and muscular strength, and reduce articular dysfunction [8] in patients with LCPD. A recent Swedish register-based study reported that physiotherapy either maintained or increased abduction [7]. Improvements in radiographic outcomes have not been reported in prospective studies [9,10] and it is not clear which activities are preferable and which should be avoided. In addition, it is unclear at what stage a return to normal activity is recommended, although some authors suggest the re-ossification stage [11]. Whether non-weightbearing and restriction of activities are effective as a treatment for LCPD in children remains controversial [12,13] and it is unclear which regime is followed in Sweden. Moreover, it is debated as to which kind of physiotherapy (stretching or strengthening exercises) is important in the treatment. Insights into the current clinical practice in Sweden may facilitate the ability to perform comparative studies, including the production of consensus guidelines.

The aim of this study was to identify the recommendations for physiotherapy, physical activity, and restricted weight-bearing when treating children at different stages of LCPD in Sweden.

## Patients and methods

### Questionnaire design

A cross-sectional survey was created in Research Electronic Data Capture (REDCap, https://www.project-redcap.org/), a web-based application for online surveys, containing a general section followed by 3 patient cases (see Supplementary data). Both quantitative and qualitative questions were included in the survey to achieve a view of the participants’ recommendations regarding physiotherapy (stretching and strengthening exercises) and physical activity in children with LCPD.

The first part of the survey collected demographic and work-related data on the participants: the number of LCPD children seen annually, years of practice as an orthopedic surgeon or physiotherapist (PT), and location of current practice. Questions on knowledge of any guidelines for physiotherapy or physical activity for children with LCPD and which factors the participant considered important when recommending physiotherapy and physical activity were also included. Factors were rated from 0 (not important) to 5 (very important) and included age at onset, ROM, sex, pain, lateral pillar classification (only for pediatric orthopedic surgeons [POSs]), and LCPD stage (see Supplementary data).

**Figure 1:**
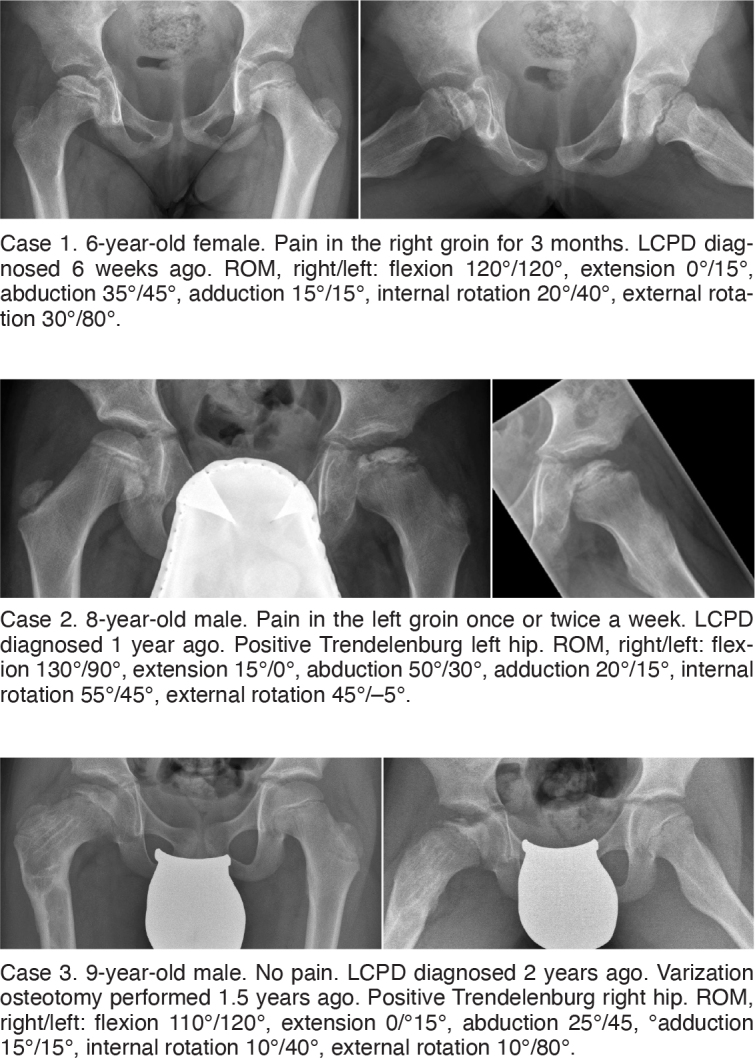
Patient cases covering the initial stage (case 1), fragmentation stage (case 2) and re-ossification stage (case 3) of Perthes disease. Frontal and frog leg/lateral radiograph and clinical characteristics. ROM = range of motion.

The second part of the survey consisted of 3 typical patient cases at different stages of the disease according to the Waldenström classification: initial stage, fragmentation stage, and re-ossification stage [14] (Figure 1).

The questions covered recommendations regarding stretching exercises, strengthening exercises, weight-bearing restrictions of the affected hip, and restrictions of physical activities for each stage of the disease. The physical activities included leisure and sports activities, such as short (< 1 km) and long walks (> 1 km), swimming, cycling, horse riding, cross-country skiing, ball sports, gymnastics, trampolining, and school sports. For each activity, participants were asked to choose between 4 options: do not allow, allow with restrictions, allow, and recommend. Each question was reviewed in detail to ensure that the questions were understandable. Furthermore, the survey underwent 2 pilot tests on different occasions to confirm high quality.

The Checklist for Reporting Of Survey Studies (CROSS) was applied.

### Study population

Members (n = 104) of the Swedish Pediatric Orthopedic Society (SBOF) and the Swedish Association of Physiotherapists as well as PTs affiliated with pediatric orthopedic departments in Sweden were invited to participate in this web survey. They received information concerning data collection, storage, and the purpose of the study, and they were asked to give consent to the publication of the survey’s results.

### Statistics

Statistical analyses were performed using SPSS version 1.0.0.1508 (IBM Corp, Armonk, NY, USA) and GraphPad Prism 9 (GraphPad Software, San Diego, CA, USA). Data was analyzed with descriptive statistical methods using means, medians, and ranges.

### Ethics, data sharing, funding, and disclosures

This study was approved by the Regional Ethical Review Board (Dnr 2021-07017-01; Sweden). The data that supports the findings of this study is available from the corresponding author (YDH) upon reasonable request. No funding was received. The authors declare no potential conflicts of interest. Completed disclosure forms for this article following the ICMJE template are available on the article page, doi: 10.2340/17453674.2023.18341

## Results

**Figure 2:**
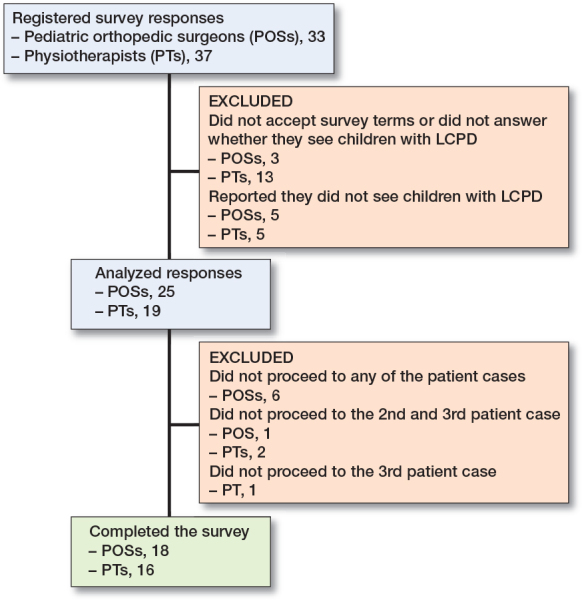
Participant flowchart.

Responses from 33 POSs and 37 PTs were registered. 3 POSs and 13 PTs did not answer the first 2 mandatory questions (survey terms and whether they see children with LCPD), and 5 POSs and 5 PTs reported that they do not see children with LCPD. Therefore, 25 responses from POSs and 19 responses from PTs were included in the analysis (Figure 2). 2 reminders were sent out to the non-responders.

20 of 25 POSs and 16 of 19 PTs reported that they treat ≤ 10 children with LCPD each year, and 2 of 25 POSs treated more than 20 patients annually. The mean work experience reported was 21 years (range 5–40 years) for POSs and 18 years (range 3–29 years) for PTs. Most of the POSs (23 of 25) were practicing in Sweden, representing 18 of the 27 hospitals that treat patients with LCPD. 1 POS was practicing in Iceland and 1 in the United Arab Emirates (both SBOF members). Two-thirds of the participants in both groups were not aware of any guidelines recommending physical activity for patients with LCPD.

### Important factors when recommending physiotherapy and physical activity

**Figure 3:**
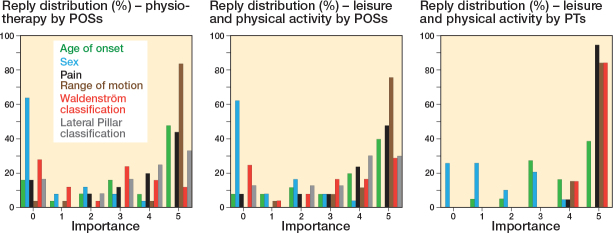
Important factors when recommending (left panel) physiotherapy by the POSs, (middle panel) leisure and physical activity by the POSs, and (right panel) leisure and physical activity by the PTs. Bars represents valid percentage of participants; 0 = not important; 5 = very important.

ROM was scored as an important factor by most of the POSs (21 of 25), followed by the age at onset (12 of 25), and the patient’s pain (11 of 25). Sex was considered as not important by 16 of the 25 participants and opinions regarding the importance of the disease stage or the Lateral Pillar classification varied. When recommending leisure and physical activities, ROM (19 of 25), patient’s pain (12 of 25), and age at onset (10 of 25) were considered important, whereas the patient’s sex was considered not important (15 of 24). Similarly, opinions concerning the importance of the disease stage or the Lateral Pillar classification varied (Figure 3).

### Recommendations for stretching and strengthening exercises

High agreement in recommending stretching exercises among the POSs was observed for all 3 patient cases; the highest score was given for the re-ossification stage (case 1, 17 of 19; case 2, 16 of 18; case 3, 17 of 18). Only 1 POS did not recommend stretching exercises at all. Most POSs agreed on offering both a home program and referral to a PT in all 3 patient cases (case, 1, 11 of 18; case 2, 12 of 17; case 3, 12 of 16). There was high agreement on not only using a home program.

**Figure 4:**
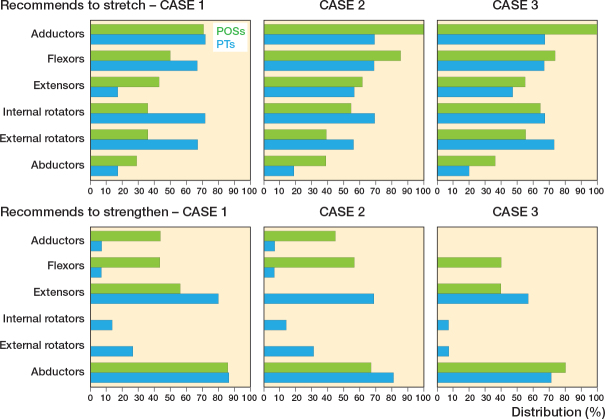
Muscle groups recommended to stretch (upper row) and strengthen (lower row) by the POSs and PTs in the initial stage (case 1), fragmentation stage (case 2), and re-ossification stage (case 3). Bars represent the valid percentage of the participants recommending training of the respective muscle group.

Strengthening exercises were prescribed with an increased trend for each disease stage (case 1, 7 of 19; case 2, 10 of 17; case 3, 15 of 18). For the initial stage, 10 of 19 POSs did not recommend strengthening exercises. Similar to the stretching exercises, most participants used both home programs and referral to a PT (case 1, 6 of 9; case 2, 8 of 11; case 3, 12 of 15). There was high agreement on not using only a home program. Muscle groups that were recommended to stretch and strengthen are presented in Figure 4.

### Weightbearing on the affected hip

For case 1 and case 3, most POSs reported weightbearing as tolerated (17 of 19 and 16 of 18, respectively) and only 2 POSs reported restricted weightbearing in each case. For the fragmentation stage (case 2), only 14 of 18 POSs reported weightbearing as tolerated, and 4 reported restricted weightbearing on the affected hip. None of the POSs recommended non-weightbearing for any of the cases.

### Leisure and sports activities

Most of the POSs did not impose any restrictions on the duration of activity (case 1, 18 of 19; case 2, 18 of 18; case 3, 16 of 17). Only 1 POS reported restrictions on the duration of activity in the initial stage, and 1 POS in the re-ossification stage.

In the initial stage, most POSs discouraged high-impact sports either completely or allowed these with restrictions. However, opinions varied on other activities. A similar pattern was seen for the fragmentation stage. For the re-ossification stage, agreement on whether to allow or restrict these activities was lower compared with the earlier 2 stages (Table).

**Table:** Answers from the pediatric orthopedic surgeons (POSs) and physiotherapists (PTs) for activities in the initial stage, fragmentation stage, and re-ossification stage. Data combined for do not allow/allow with restrictions (no) and allow/recommend (yes). Values are count

Factor	Initial stage				Fragmentation stage				Re-ossification stage			
	POS		PT		POS		PT		POS		PT	
	No	Yes	No	Yes	No	Yes	No	Yes	No	Yes	No	Yes
Short walks (< 1 km)	3	16	3	14	2	16	3	12	0	18	0	15
Long walks (> 1 km)	6	10	15	2	11	6	14	1	4	14	6	9
Swimming	1	17	0	18	1	17	0	16	0	18	0	15
Cycling	2	17	0	18	3	15	0	16	2	16	0	15
Horse riding	3	15	1	15	4	14	1	13	2	16	1	13
Cross-country skiing	7	11	7	9	7	9	7	8	4	14	2	12
Ice skating	8	10	11	6	9	9	7	8	4	14	4	10
Kick bike/inline	11	6	10	8	13	5	7	8	5	13	4	11
Dancing	12	7	14	3	11	6	12	3	5	12	6	9
Running	16	3	16	1	14	4	14	1	9	9	9	6
Table tennis	6	12	13	5	9	8	10	6	4	13	5	10
Ball sports	17	2	16	1	14	2	13	2	8	10	9	4
Alpine skiing	14	4	15	1	13	4	14	1	7	10	8	4
Gymnastics	15	4	16	1	14	3	14	1	7	11	9	6
Trampolining	17	2	18	0	16	2	16	0	10	7	12	3
School sports	11	7	16	2	13	5	14	2	8	10	7	8

There was high agreement on recommending low-impact sports. Opinions varied on returning to other activities. Some POSs remained cautious but others either allowed or even recommended these activities. The number of years in practice of the POSs showed no impact on the recommendations or restrictions given.

### Physiotherapists

In contrast to the POSs, PTs rated the patient’s pain as the most important factor when prescribing physiotherapy, followed by LCPD stage and ROM. Opinions varied regarding age at onset and sex (Figure 3).

In accordance with the POSs, there was high agreement in recommending stretching exercises for all 3 patient cases. Most of the PTs recommended stretching at least once a day, and in the reossification stage half of the PTs proposed at least once a day and a third at least 3 times a week. PTs recommended strengthening exercises to a greater extent than POSs, mainly in the initial stage. Most of the PTs proposed strengthening exercises at least 2 to 3 times a week.

In contrast to the POSs, higher consensus was found among PTs in restricting activities in the initial and fragmentation stages. In the re-ossification stage, agreement among PTs was higher than among POSs in restricting activities such as running, ball sports, alpine skiing, gymnastics, and trampolining.

Activities that were allowed or recommended for the initial and fragmentation stages included swimming, cycling, horse riding and short walks, in agreement with the POSs. In addition, cross-country skiing and skating were approved for the re-ossification stage by both groups.

PTs were asked about a recommendation on assistive devices. Mostly crutches and wheelchairs were prescribed in all 3 cases. Other devices mentioned were walking aids, walking frames, bicycles, and strollers. For case 3, more than half would not prescribe any assistive device.

## Discussion

The aim of this survey was to gain valuable insight into the current recommendations for physiotherapy, physical activity, and weightbearing among POS and PTs when treating children at different stages of LCPD in Sweden. The study showed that ROM was considered to be important when recommending physical activity. Our study showed some inconsistency within professions and between POSs and PTs. This may be explained by different experiences and beliefs that might be important for the patient.

Sex was reported as unimportant when recommending physiotherapy or leisure and sports activities, suggesting that participants do not treat girls differently, even though female sex has been shown to be associated with a worse outcome [10]. ROM in contrast was considered very important, probably with the goal to maintain or improve mobility [7,8] which is essential before surgery [15]. Age was another factor that was considered important when recommending physiotherapy, probably because the long-term outcome is age-associated [10,16]. The opinion on the importance of the Lateral Pillar classification when recommending physiotherapy varied, even though the ROM seems to decrease with increased engagement of the lateral pillar [7]. A possible explanation could be that most patients in Sweden are diagnosed at an early stage [7] when the Lateral Pillar classification is not applicable.

Stretching was recommended by the participants for all 3 disease stages, which is in line with the survey by the British Society for Children’s Orthopaedic Surgery [17]. In Sweden, previous results from the Swedish Pediatric Orthopedic Quality Register showed that 63% either received instructions on abduction training or were referred to a PT [7].

Whether the strengthening exercises should be included early is not established in the literature. However, we found high agreement in recommending strengthening exercises first in the re-ossification stage with a special focus on the abductor muscles.

Interestingly, there was 100% agreement in not recommending strict non-weightbearing of the affected hip, indicating a united approach by the participants on a matter that remains controversial internationally [1,3]. This is in line with the idea that prolonged immobilization fails to alter the radiographic course of the disease [13], and weightbearing and activity restriction in the active stages of Perthes disease were associated with worse patient-reported mobility scores [18]. Conversely, weight-bearing caused larger dynamic deformation of the femoral head with LCPD compared with the non-affected side in an MRI study [6] and non-weightbearing has been reported to favor decreased deformation of the femoral head [19]. However, patients and parents are interested in the long-term consequences, such as pain, ROM, ability to participate in sports, or risk of premature osteoarthritis [20]. Neither of these factors has been taken into consideration when recommending weightbearing restrictions as most studies are based only on short-term radiographic outcomes. Furthermore, none of these studies has taken the children’s compliance into consideration, which has been shown low in adult patients after fractures [21-23]. Recommending prolonged hip unloading using crutches or a wheelchair might be very challenging in children, both psychologically and socially [24], especially in a patient group with associated attention deficit hyperactivity disorders (ADHD) [25].

We showed inconsistencies regarding precautions, which might be problematic for both patients and parents as shown by Leo et al. [25], i.e. that the parents wish for more guidance on physical activity. Full activity in the late re-ossification stage seems reasonable since MRI studies have shown less deformation when weightbearing is applied in the later stages of LCPD [12] when normal bone texture is found [26].

### Limitations

The response rate was difficult to determine because the survey was sent to all members of the professional societies (Swedish Pediatric Orthopedic Society and the Swedish Association of Physiotherapists). However, not all members treat pediatric patients or patients with LCPD specifically. The answers received were geographically dispersed, which implies that the participants with a special interest in treating patients with LCPD were represented by those hospitals with the competence to treat LCPD. In addition, most participants had long work experience, with none less than 5 years, and only 6 POSs with less than 12 years of work experience. Another caveat is the choice of cases that differed in several characteristics such as age, sex, disease stage, and clinical presentation, including surgery. Even though we intended to present typical cases, it may still be difficult to draw final conclusions based on these examples.

### Conclusion

When recommending physical therapy, ROM is considered to be important; PTs also recognize patients’ pain and disease stage as important. We found high agreement in recommending stretching exercises for all disease stages, but there are controversies regarding recommendations for strengthening exercises for the initial and fragmentation stages. Further, non-weightbearing treatment for the affected hip was not recommended at any stage of the disease but high consensus was found in discouraging high-impact activities in the initial and fragmentation stage and allowing swimming, short walks, cycling, and horse riding without restrictions for all stages. There was no clear consensus regarding the appropriate timeline for resuming full activities.

The study confirms the necessity to identify which exercises and activities are the most beneficial for each stage and to give clear and consistent recommendations to children and parents. National and international guidelines should be created to ensure equal healthcare for all children with LCPD.

### Supplementary data

The questionnaire distributed to pediatric orthopedic surgeons and physiotherapists is available on the article homepage, doi: 10.2340/17453674.2023.18341

## Supplementary Material

Recommendations for physiotherapy and physical activity for children with Legg–Calvé–Perthes disease: a survey of pediatric orthopedic surgeons and physiotherapists in Sweden

## Notes

YDH planned the study and made substantial contributions to the design, the web-based questionnaire, data collection, and interpretation, and did a critical revision of the manuscript. LM participated in the design of the study and the web-based questionnaire, data collection, data analysis, statistics and interpretation, and drafting of the manuscript. ZR contributed to data analysis, statistics, and drafting of the manuscript.

The authors would like to thank all participants for sharing their valuable time and experience in this survey.

Handling co-editor: Ivan Hvid

*Acta* thanks Vilhelm Engell and Martin Gottliebsen for help with peer review of this manuscript.
