# Supplementary material for: Recommendations for physiotherapy and physical activity for children with Legg–Calvé–Perthes disease: a survey of pediatric orthopedic surgeons and physiotherapists in Sweden
Source: Acta Orthop. 2023 Aug 16;94:432–7. doi: 10.2340/17453674.2023.18341 (PMC10436286; doi:10.2340/17453674.2023.18341)
Supplement: Recommendations for physiotherapy and physical activity for children with Legg–Calvé–Perthes disease: a survey of pediatric orthopedic surgeons and physiotherapists in Sweden [file ActaO-94-18341-s001.pdf]

Legg-Calvé Perthes and physical activity: recommendations and clinical use among pediatric orthopedic surgeons.

Dear participant,

We would like to invite you to participate in a research study regarding recommendations for physiotherapy and physical activity in patient with ongoing Legg-Calvé Perthes Disease (LCPD). This survey aims to identify how and to what extent pediatric orthopedic surgeons currently prescribe physiotherapy when treating children with LCPD in different stages of the disease. The purpose is to analyze similarities and differences in current clinical practice.

If you do not see or treat children with Perthes disease we would appreciate your participation. The survey will end after the first question.

The survey consists of four parts: one general part followed by three cases covering the initial stage, fragmentation stage and re-ossification stage of Perthes disease. The survey should take approximately 10-20 minutes to answer.

The data will be collected and stored through REDCap

The survey is in English since it will be used for international participants as well. However, you can answer in English or in Swedish.

Best regards,
Yasmin Hailer, Pediatric orthopedic surgeon, Akademiska sjukhuset, Uppsala
Louise Melin, Medical student, Uppsala University

Information

Please read the information above before entering the survey

I have read the information and accept the terms for collection, storage and use of my participation in this survey.	◯ Yes<br>◯ No

General questions

Do you see children with Legg-Calvé Perthes disease?

◯ Yes
◯ No

How many new and follow-up Perthes patients do you see each year?

◯ 1-5
◯ 6-10
◯ 11-15
◯ 16-20
◯ >20

Are you an orthopedic surgeon or an orthopedic resident?

◯ Orthopedic surgeon
◯ Orthopedic resident

How many years have you practiced as an orthopedic surgeon?

____
((years))

Which country do you currently practice in?

____
((country))

What city do you currently practice in?

____
((city))

Are you aware of any physical activity guidelines for surgeons recommending physical activity to patients with Perthes disease?

◯ Yes
◯ No

Please specify which ones:

____

Which of following factors do you consider when recommending physiotherapy for your patients with Perthes disease? Please rate from 0-5. (0: not important, 5: very important)

	0 (not important)	1	2	3	4	5 (very important)	I prefer not to answer
Age of onset	◯	◯	◯	◯	◯	◯	◯
Sex	◯	◯	◯	◯	◯	◯	◯
Patient's pain	◯	◯	◯	◯	◯	◯	◯
Range of motion	◯	◯	◯	◯	◯	◯	◯
Waldenströms classification	◯	◯	◯	◯	◯	◯	◯
Lateral Pillar classification	◯	◯	◯	◯	◯	◯	◯

If other: Please specify

____

Which of following factors do you consider when recommending leisure and physical activity for your patients with Perthes? Please rate from 0-5 (0: not important, 5: very important)

	0 (not important)	1	2	3	4	5 (very important)	I prefer not to answer
Age of onset	◯	◯	◯	◯	◯	◯	◯
Sex	◯	◯	◯	◯	◯	◯	◯
Patient's pain	◯	◯	◯	◯	◯	◯	◯
Range of motion	◯	◯	◯	◯	◯	◯	◯
Waldenströms classification	◯	◯	◯	◯	◯	◯	◯
Lateral Pillar classification	◯	◯	◯	◯	◯	◯	◯

If other: Please specify

____

Initial stage

You will now be presented three cases covering initial stage, fragmentation stage and re-ossification stage.

Case 1: 6 year old female. Pain in the right groin for 3 months. Perthes disease diagnosed 6 weeks ago.

ROM in degree
Flexion right vs left: 120 vs 120
Extension right vs left: 0 vs 15
Abduction right vs left: 35 vs 45
Adduction right vs left: 15 vs 15
Internal rotation right vs left: 20 vs 40
External rotation right vs left: 30 vs 80

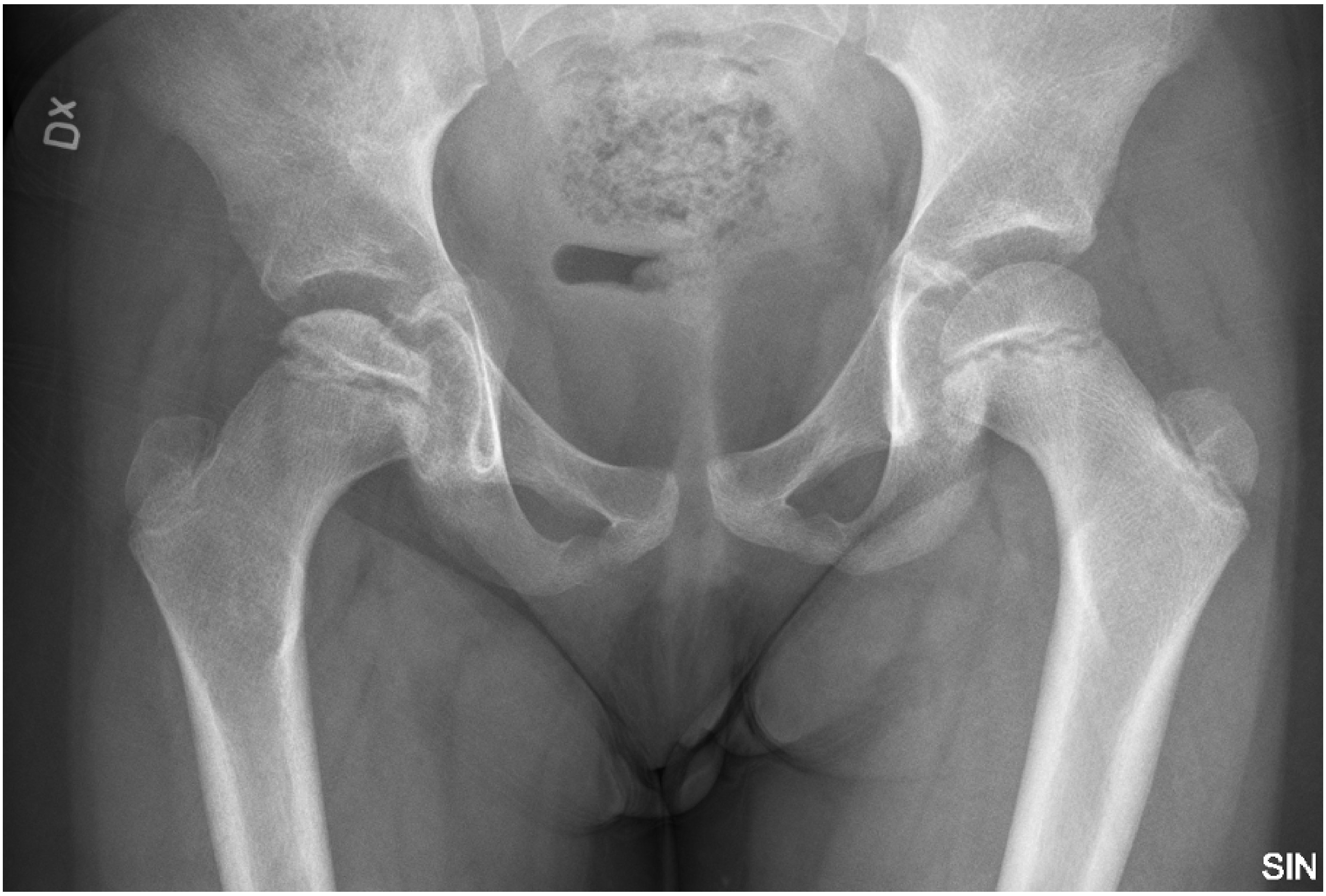

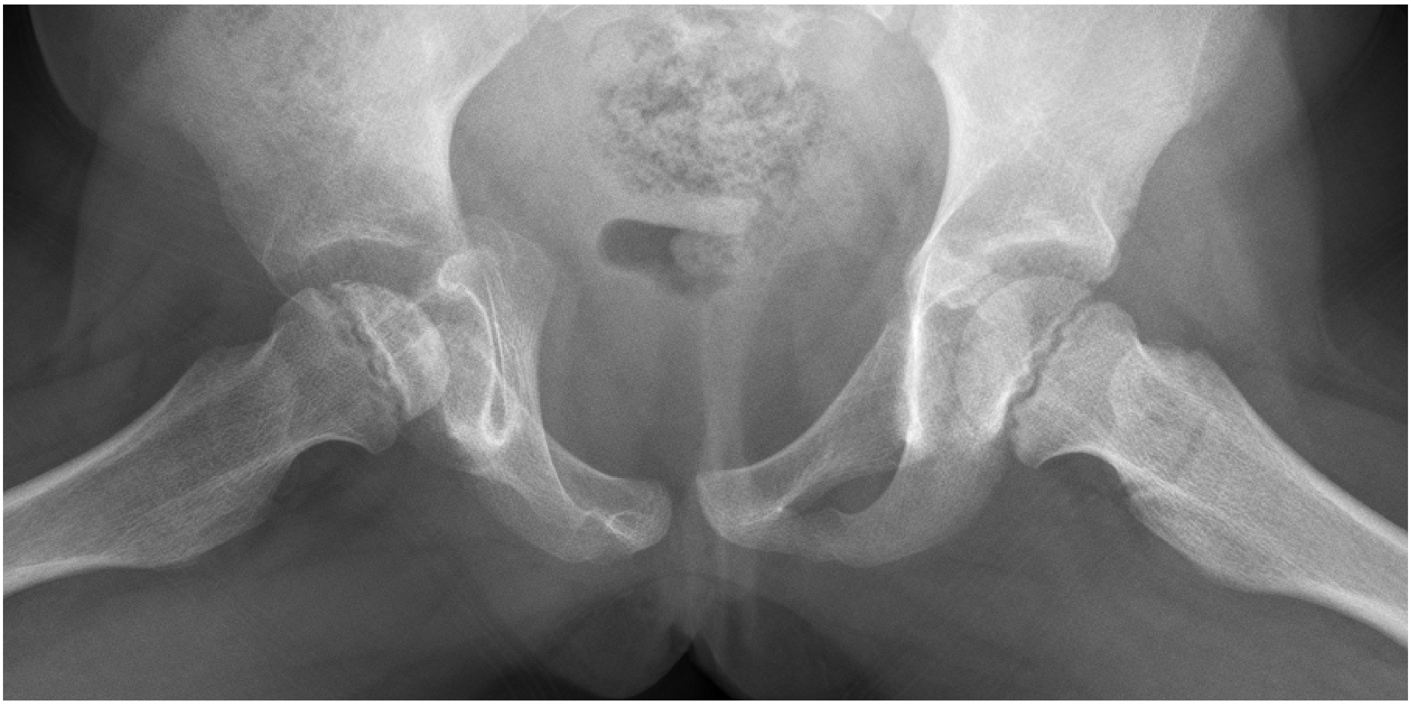

Do you recommend stretching exercises for this patient?

- ◯ Yes, always
- ◯ Only if...
- ◯ No
- ◯ I prefer not to answer this question

Please specify

____

For which muscle groups would you recommend stretching?

____

How do you recommend this patient to perform these exercises?

- ◯ Home program and information to parents
- ◯ Referral to a physiotherapist
- ◯ Both home program and physiotherapist
- ◯ Other

If other: Please specify

____

Do you recommend strengthening exercises for this patient?

- ◯ Yes, always
- ◯ Only if...
- ◯ No
- ◯ I prefer not to answer this question

Please specify

____

For which muscle groups would you recommend strengthening?

____

How do you recommend this patient to perform these exercises?

- ◯ Home program and information to parents
- ◯ Referral to a physiotherapist
- ◯ Both home program and physiotherapist
- ◯ Other

If other: Please specify

____

How would you recommend weight-bearing of the affected hip?

- ◯ Weight-bearing as tolerated
- ◯ Restricted weight-bearing
- ◯ No weight-bearing

Please specify your weight-bearing restrictions.

____

What are your recommendations for the following leisure or sport activities for this patient?
Please rate between: Do not allow - Allow with restrictions - Allow - Recommend

	Do not allow	Allow with restrictions	Allow	Recommend	I prefer not to answer
Short walks (< 1 km)	◯	◯	◯	◯	◯
Long walks (>1 km)	◯	◯	◯	◯	◯
Swimming	◯	◯	◯	◯	◯
Cycling	◯	◯	◯	◯	◯
Horse-riding	◯	◯	◯	◯	◯
Cross-country skiing	◯	◯	◯	◯	◯
Ice skating	◯	◯	◯	◯	◯
Kickbike/inline-skating	◯	◯	◯	◯	◯
Dancing	◯	◯	◯	◯	◯
Running	◯	◯	◯	◯	◯
Table-tennis	◯	◯	◯	◯	◯
Ball sports (tennis, football, basket)	◯	◯	◯	◯	◯
Skiing (alpine)	◯	◯	◯	◯	◯
Gymnastics	◯	◯	◯	◯	◯
Trampoline	◯	◯	◯	◯	◯
School sport	◯	◯	◯	◯	◯

You have selected one or more activities above which you allow with restrictions. Could you please specify your restrictions

____

Do you place any restrictions concerning time of activity?

- ◯ Yes
- ◯ No

What maximum time of activity do you find acceptable?

____

Any further comments regarding this stage of the disease and leisure or sport activities? Please elaborate.

____

Fragmentation stage

Case 2: 8 year old male. Pain in the left groin once or twice a week. Perthes diseases diagnosed 1 year ago. Positive Trendelenburg left hip

ROM in degree
Flexion right vs left: 130 vs 90
Extension right vs left: 15 vs 0
Abduction right vs left: 50 vs 30
Adduction right vs left: 20 vs 15
Internal rotation right vs left: 55 vs 45
External rotation right vs left: 45 vs -5

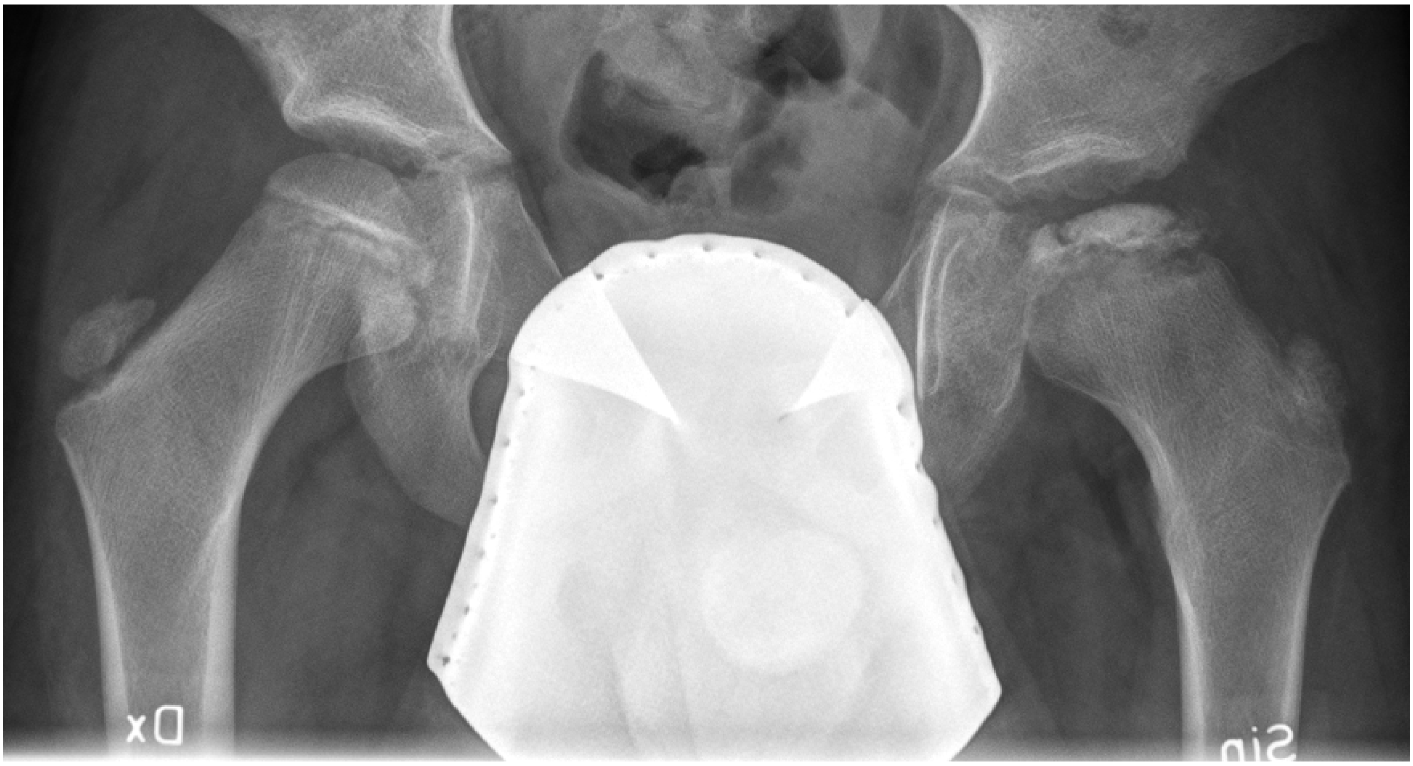

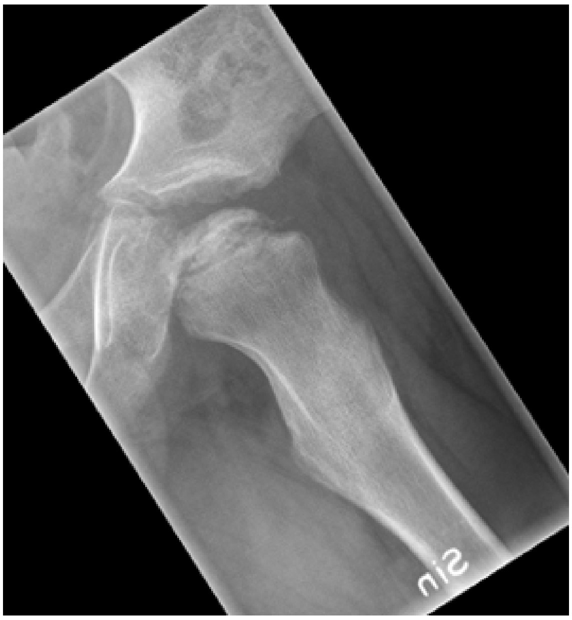

Do you recommend stretching exercises for this patient?	◯ Yes, always<br>◯ Only if...<br>◯ No<br>◯ I prefer not to answer this question
Please specify	__________________________________
For which muscle groups would you recommend stretching?	__________________________________
How do you recommend this patient to perform these stretching exercises?	◯ Home program and information to parents<br>◯ Referral to a physiotherapist<br>◯ Both home program and physiotherapist<br>◯ Other
If other: Please specify	__________________________________
Do you recommend strengthening exercises for this patient?	◯ Yes, always<br>◯ Only if...<br>◯ No<br>◯ I prefer not to answer this question
Please specify	__________________________________
For which muscle groups would you recommend strengthening?	__________________________________
How do you recommend this patient to perform these exercises?	◯ Home program and information to parents<br>◯ Referral to a physiotherapist<br>◯ Both home program and physiotherapist<br>◯ Other
If other: Please specify	__________________________________
How would you recommend weight-bearing of the affected hip?	◯ Weight-bearing as tolerated<br>◯ Restricted weight-bearing<br>◯ No weight-bearing
Please specify your weight-bearing restrictions	__________________________________

What are your recommendations for the following leisure or sport activities for this patient?
Please rate between: Do not allow - Allow with restrictions - Allow - Recommend

	Do not allow	Allow with restrictions	Allow	Recommend	I prefer not to answer
Short walks (< 1 km)	○	○	○	○	○
Long walks (>1 km)	○	○	○	○	○
Swimming	○	○	○	○	○
Cycling	○	○	○	○	○
Horse-riding	○	○	○	○	○
Cross-country skiing	○	○	○	○	○
Ice skating	○	○	○	○	○
Kickbike/inline-skating	○	○	○	○	○
Dancing	○	○	○	○	○
Running	○	○	○	○	○
Table-tennis	○	○	○	○	○
Ball sports (tennis, football, basket)	○	○	○	○	○
Skiing (alpine)	○	○	○	○	○
Gymnastics	○	○	○	○	○
Trampoline	○	○	○	○	○
School sport	○	○	○	○	○

You have selected one or more activities above which you allow with restrictions. Could you please specify your restrictions.

____

Do you place any restrictions concering time of activity?

○ Yes
○ No

What maximum time of activity do you find acceptable?

____

Any further comments regarding this stage of the disease and leisure or sport activities? Please elaborate.

____

Re-ossification stage

Case 3: 9 year old male. No pain. Perthes disease diagnosed 2 years ago. Varisation osteotomy 1,5 years ago.
Positive Trendelenburg right hip

ROM in degree
Flexion right vs left: 110 vs 120
Extension right vs left: 0 vs 15
Abduction right vs left: 25 vs 45
Adduction right vs left: 15 vs 15
Internal rotation right vs left: 10 vs 40
External rotation right vs left: 10 vs 80

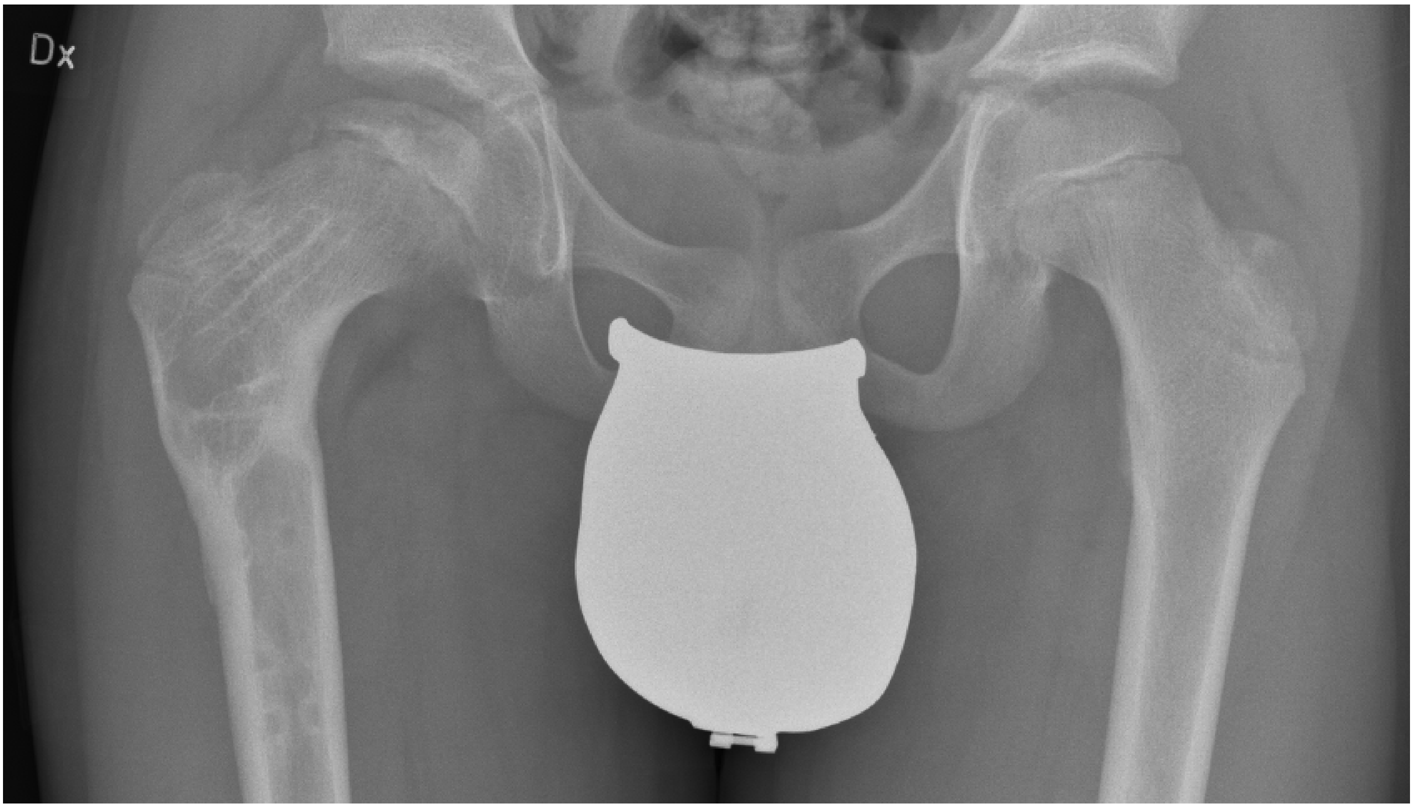

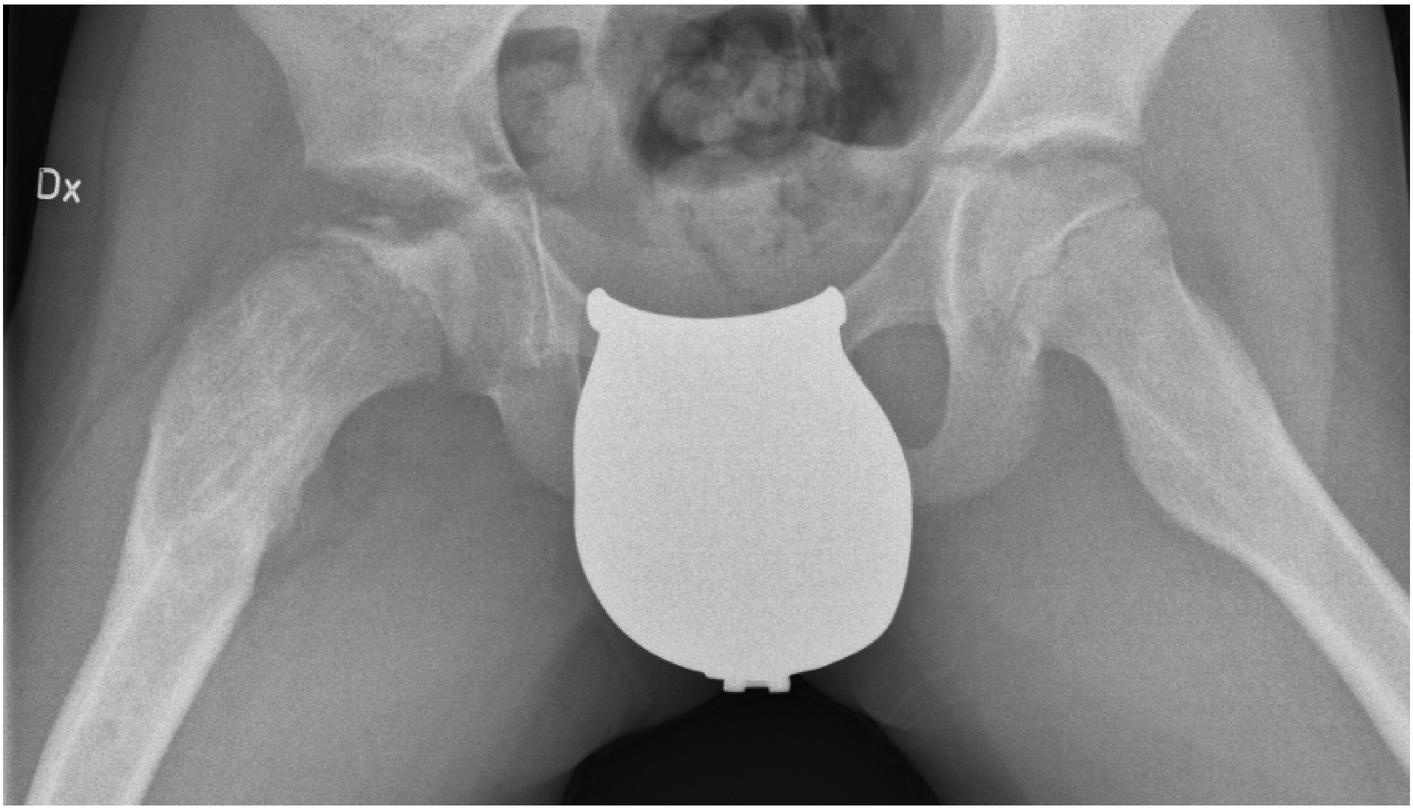

Do you recommend stretching exercises for this patient?	◯ Yes, always<br>◯ Only if...<br>◯ No<br>◯ I prefer not to answer this question
Please specify	__________________________________
For which muscle groups would you recommend stretching?	__________________________________
How do you recommend this patient to perform these stretching exercises?	◯ Home program and information to parents<br>◯ Referral to a physiotherapist<br>◯ Both home program and physiotherapist<br>◯ Other
If other: Please specify	__________________________________
Do you recommend strengthening exercises for this patient?	◯ Yes, always<br>◯ Only if...<br>◯ No<br>◯ I prefer not to answer this question
Please specify	__________________________________

For which muscle groups would you recommend strengthening?

____

How do you recommend this patient to perform these exercises?

- ◯ Home program and information to parents
- ◯ Referral to a physiotherapist
- ◯ Both home program and physiotherapist
- ◯ Other

If other: Please specify

____

How would you recommend weight-bearing of the affected hip?

- ◯ Weight-bearing as tolerated
- ◯ Restricted weight-bearing
- ◯ No weight-bearing

Please specify your weight-bearing restrictions

____

What are your recommendations for the following leisure or sport activities for this patient?
Please rate between: Do not allow - Allow with restrictions - Allow - Recommend

	Do not allow	Allow with restrictions	Allow	Recommend	I prefer not to answer
Short walks (< 1 km)	◯	◯	◯	◯	◯
Long walks (>1 km)	◯	◯	◯	◯	◯
Swimming	◯	◯	◯	◯	◯
Cycling	◯	◯	◯	◯	◯
Horse-riding	◯	◯	◯	◯	◯
Cross-country skiing	◯	◯	◯	◯	◯
Ice skating	◯	◯	◯	◯	◯
Kickbike/inline-skating	◯	◯	◯	◯	◯
Dancing	◯	◯	◯	◯	◯
Running	◯	◯	◯	◯	◯
Table-tennis	◯	◯	◯	◯	◯
Ball sports (tennis, football, basket)	◯	◯	◯	◯	◯
Skiing (alpine)	◯	◯	◯	◯	◯
Gymnastics	◯	◯	◯	◯	◯
Trampoline	◯	◯	◯	◯	◯
School sport	◯	◯	◯	◯	◯

You have selected one or more activities above which you allow with restrictions. Could you please specify your restrictions.

____

Do you place any restrictions concerning time of activity?

- ◯ Yes
- ◯ No

What maximum time of activity do you find acceptable?

____

Any further comments regarding this stage of the disease and leisure or sport activities? Please elaborate.

____
